# Functional assessment of von Willebrand factor expression by cancer cells of non-endothelial origin

**DOI:** 10.18632/oncotarget.14273

**Published:** 2016-12-27

**Authors:** Anahita Mojiri, Konstantin Stoletov, Maria Areli Lorenzana Carrillo, Lian Willetts, Saket Jain, Roseline Godbout, Paul Jurasz, Consolato M. Sergi, David D. Eisenstat, John D. Lewis, Nadia Jahroudi

**Affiliations:** ^1^ Department of Medicine, University of Alberta, Edmonton, Alberta, Canada; ^2^ Department of Oncology, University of Alberta, Edmonton, Alberta, Canada; ^3^ Department of Pharmacy and Pharmaceutical Sciences, University of Alberta, Edmonton, Alberta, Canada; ^4^ Department of Laboratory Medicine and Pathology, University of Alberta, Edmonton, Alberta, Canada; ^5^ Departments of Medical Genetics and Pediatrics, University of Alberta, Edmonton, Alberta, Canada

**Keywords:** von Willebrand factor, cancer, extravasation, transcription, transcription factor

## Abstract

Von Willebrand factor (VWF) is a highly adhesive procoagulant molecule that mediates platelet adhesion to endothelial and subendothelial surfaces. Normally it is expressed exclusively in endothelial cells (ECs) and megakaryocytes. However, a few studies have reported VWF detection in cancer cells of non-endothelial origin, including osteosarcoma. A role for VWF in cancer metastasis has long been postulated but evidence supporting both pro- and anti-metastatic roles for VWF has been presented. We hypothesized that the role of VWF in cancer metastasis is influenced by its cellular origin and that cancer cell acquisition of VWF expression may contribute to enhanced metastatic potential. We demonstrated *de novo* expression of VWF in glioma as well as osteosarcoma cells. Endothelial monolayer adhesion, transmigration and extravasation capacities of VWF expressing cancer cells were shown to be enhanced compared to non-VWF expressing cells, and were significantly reduced as a result of VWF knock down. VWF expressing cancer cells were also detected in patient tumor samples of varying histologies. Analyses of the mechanism of transcriptional activation of the VWF in cancer cells demonstrated a pattern of trans-activating factor binding and epigenetic modifications consistent overall with that observed in ECs. These results demonstrate that cancer cells of non-endothelial origin can acquire *de novo* expression of VWF, which can enhance processes, including endothelial and platelet adhesion and extravasation, that contribute to cancer metastasis.

## INTRODUCTION

Von Willebrand Factor (VWF) is a procoagulant protein with an expression pattern that is highly restricted to endothelial cells (ECs) and megakaryocytes, and commonly used as a marker of ECs [1]. It functions as a mediator of platelet-endothelial/subendothelial adhesion to promote platelet aggregate formation, as well as a carrier for Factor VIII in the circulation [2–5]. In addition to its major role in hemostasis, VWF has been reported to participate in the immune response, inflammation, angiogenesis and cancer metastasis [6–8].

Regarding the role of VWF in cancer metastasis, it was hypothesized that VWF participates in adhesion of cancer cells to platelets and endothelial surfaces, thus facilitating extravasation and promoting metastasis [9]. A role for platelets in cancer metastasis is well established. Tumor cells associate with platelets in the circulation and form heteroaggregates, which is proposed to either protect tumor cells from immune surveillance thus increasing their circulatory half-life, and/or contribute to the metastatic process through association of heteroaggregates with the vascular endothelium [10, 11]. Thus, VWF, as a major participant in promoting platelet aggregation/endothelium interactions, presents itself as a highly likely candidate to promote metastasis. Consistent with this hypothesis, anti-VWF antibodies were shown to decrease metastatic activities of some cancer cell lines passaged as xenotransplants in mice, and inhibited adhesion of a colon cancer cell line to ECs in a co-culture adhesion assay [12, 13]. Additionally, VWF fibers in tumor vasculature were shown to mediate platelet aggregation and contribute to melanoma metastasis [14]. In contrast, analyses of tumor cell metastasis in VWF deficient mice clearly demonstrated that VWF deficiency significantly enhanced tumor metastasis [7], thus presenting VWF as an anti-metastatic protein. Consistent with these results, further investigations demonstrated that VWF exerts a pro-apoptotic effect on the tumor cells, thus leading to tumor cell death and consequently reduced metastasis [15, 16]. However, clinical studies exploring levels of VWF in cancer patients, and specifically those with von Willebrand disease (VWD), have presented a picture that is more consistent with a pro-metastatic role for VWF [9]. Increased levels of plasma VWF have been consistently demonstrated in patients with colorectal, breast, prostate, ovarian, and other types of malignancies. Furthermore higher VWF levels were detected in cancers with distant metastasis [9, 17–19].

Discordant observations regarding the role of VWF in cancer metastasis may be, at least partly, attributed to the focus of investigations on ECs and platelets as the source of VWF. Since VWF expression was long believed to be an exclusive property of these two cell types, the possibility that VWF may also be expressed in cancer cells of non-endothelial origin has been generally unexplored. However, there have been a few reports of VWF detection in cancer cells of non-endothelial origin. VWF protein was reported in cultured human osteosarcoma SAOS2 cells, human colorectal SW480 cancer cells, and recently in two hepatocellular carcinoma cell lines HepG2 and BEL7402 [20–22]. In these studies, increased levels of VWF in osteosarcoma and hepatocellular carcinoma tumor tissues *in situ* were demonstrated and associated with increased metastasis and clinicopathologic staging [20, 21]. Increased VWF levels were not associated with increased vascular density [20], suggesting that increased VWF expression may have a cellular origin that is distinct from vascular ECs. Based on these reports, we explored whether some cancer cells of non-endothelial origin, including glioma as well as osteosarcoma SAOS2, acquire *de novo* transcription of the VWF gene and determined the functional consequences with regard to tumor cell adhesion and extravasation. We also explored alterations in transcriptional regulatory mechanisms that are associated with activation of the VWF gene transcription in cancer cells, and also demonstrated presence of VWF expressing cancer cells in patient's tumor samples of glioma and osteosarcoma. These results demonstrated that cancer cells that acquire *de novo* VWF expression have increased endothelium adhesion and extravasation potential, which is conducive to increased metastasis.

## RESULTS

### VWF is expressed in cancer cells of non-endothelial cell origin

**Figure 1:**
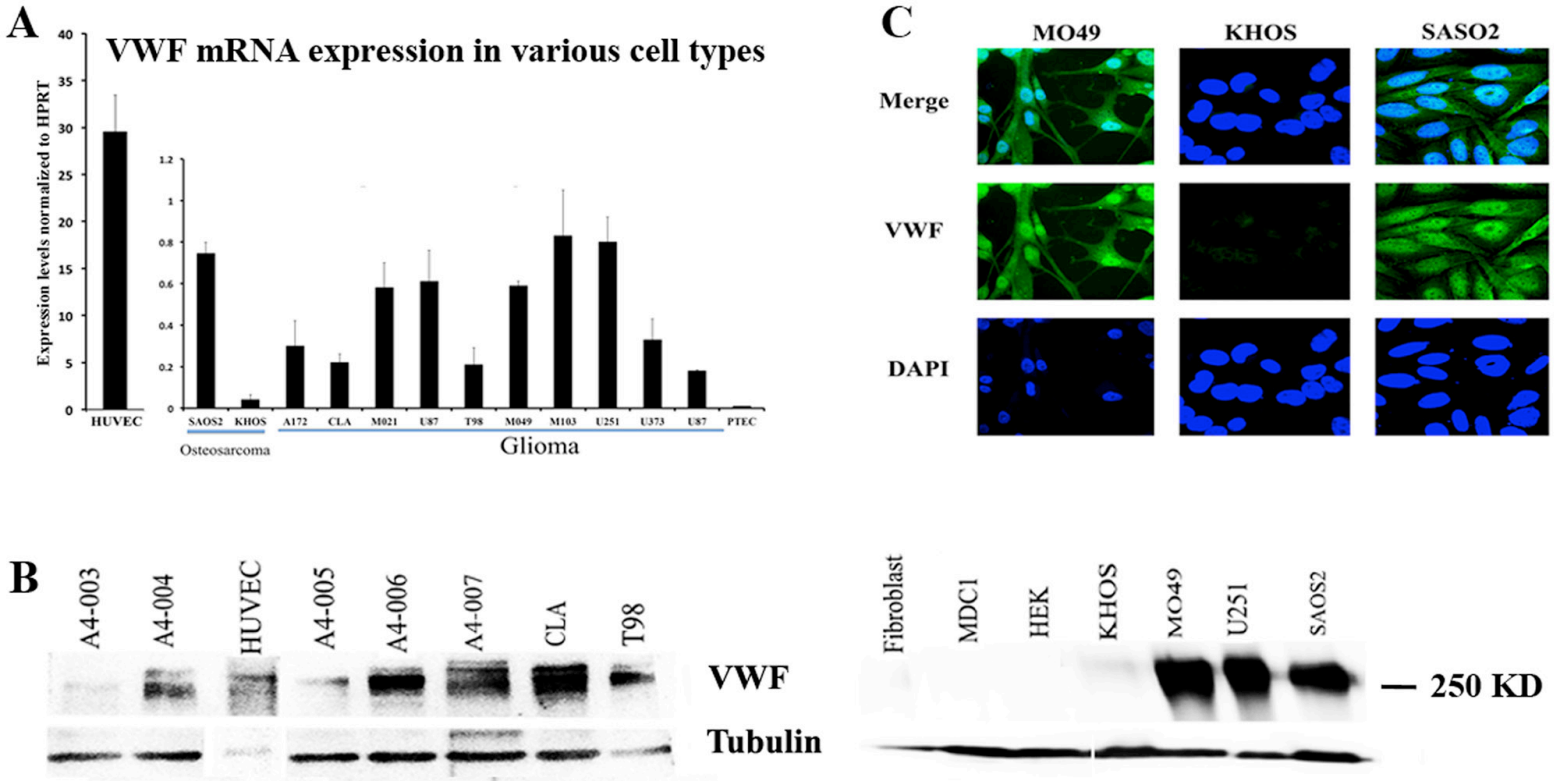
VWF is expressed in some cancer cell lines of non-endothelial origin **(A)** Quantitative RT-PCR analyses were performed to detect VWF mRNA expression in osteosarcoma cell lines SAOS2 and KHOS as well as several malignant glioma cell lines (on the chart from A172 to U87). Proximal tubular epithelial cells (PTEC) were used as a negative control. Human umbilical vein endothelial cells (HUVEC) were used as positive control and presented with separate Y axis scale demonstrating significantly higher levels of VWF mRNA in comparison to that detected in cancer cells. The levels of VWF mRNA were normalized to HPRT. **(B)** Western blot analysis using human VWF specific antibody was performed to detect VWF protein. Cell lysates from two osteosarcoma cell lines SAOS2 and KHOS, several malignant glioma cell lines [those used for RNA analysis (M049 and U251, CLA, T98)], several patient derived glioblastoma cells (A4-003 to A4-007), several other non-endothelial cell types (used as negative controls) including HEK 293 (HEK), human primary fibroblasts (Fibroblast) and primary dendritic cells (MDC1), as well as HUVEC (positive control) were used for these analyses. Tubulin expression was used as a loading control. Due to significantly higher levels of VWF expression in HUVECs the total protein loaded from these cells was reduced compared to other cell types, as shown by lower levels of tubulin. The positive control from HUVEC serves to demonstrate the expected position of VWF at 250 KD. **(C)** KHOS, SAOS2 and glioma M049 cell lines were subjected to immunofluorescence staining to detect VWF (green). DAPI staining (blue) marked the nucleus (20X magnification). Results are representative of 3-4 independent experiments.

To determine whether VWF is expressed in cancer cells, we screened a variety of malignant glioma cell lines, including those prepared from patient-derived glioblastoma tumor samples, as well as two osteosarcoma cell lines SAOS2 and KHOS to detect VWF mRNA and protein. Various levels of VWF mRNAs were detected by quantitative RT-PCR in malignant glioma and SAOS2 cell lines, but not in any detectable levels in KHOS, or proximal tubule epithelial cells (PTEC) used as negative control (Figure 1A). As expected, levels of expression from VWF expressing cancer cells were significantly lower than that expressed by human umbilical vein endothelial cells (HUVECs), which are the cell types that normally express VWF. Expression of VWF at the protein level was detected by Western blot analysis in selected malignant glioma cancer cells (those used in RNA analyses), as well as other patient tumor-derived glioblastoma cancer cells (A4-003 to A4-007), and also in SAOS2, and HUVEC (positive control), but not in KHOS or other primary and established cell lines of non-endothelial origin that were used as negative controls (Figure 1B). VWF expression was also demonstrated by immunofluorescence staining in SAOS2 and a representative patient derived malignant glioma cell line M049, but not in KHOS (Figure 1C). These results demonstrated that some cancer cells of non-endothelial origin express VWF at the RNA and protein levels. VWF expression appeared throughout the cells and also covered the nuclear region but this may be in the cytoplasmic region overlying the nucleus and from these analyses we cannot confirm or exclude nuclear localization in these cells.

### Functional consequences of VWF expression by cancer cells regarding endothelial monolayer and platelet adhesion

**Figure 2:**
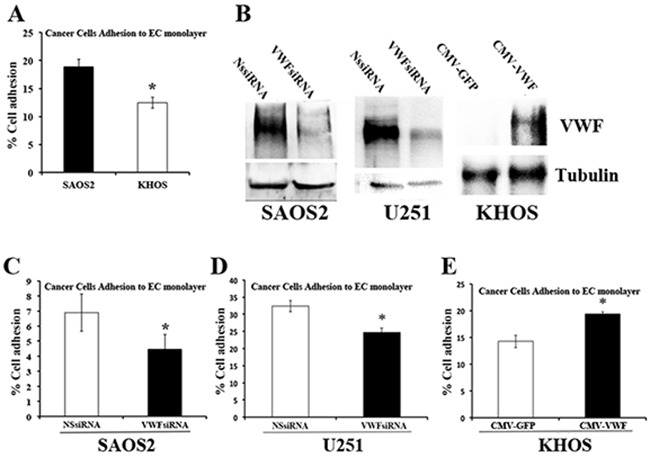
VWF expression increases the adhesion capacity of cancer cells to endothelial monolayer **(A)** Adhesion assay was performed, as described in Methods, on SAOS2 and KHOS that were labelled with fluorescent CellTracker™ Green and incubated on HUVEC monolayer. Percentages of adherent cells were determined by flow cytometry. **(B)** Western blot analyses demonstrating VWF expression in SAOS2 and U251 that were treated with VWF specific (VWFsiRNA) or non specific (NSsiRNA) siRNAs, and KHOS cells that were transduced with control lentivirus (CMV-GFP) or VWF expressing lentivirus (CMV-VWF). **(C-E)** Adhesion assays were performed as described for **(A)** on SAOS2 and U251 cells that were transfected with non-specific siRNA (NSsiRNA) or VWF specific siRNA (VWFsiRNA), and KHOS cells transduced with lentiviral vectors harboring CMV-GFP or CMV-VWF.

To determine whether VWF expression influences the ability of cancer cells to adhere to endothelium, VWF expressing (SAOS2) and non-expressing (KHOS) osteosarcoma cell lines were treated with cytoplasmic staining CellTracker™ Green for visualization, and equal numbers of cells were incubated on the monolayer of ECs. Adhesion of cancer cells to endothelial monolayers was determined by IF staining and FACS analyses, as described in Methods. SAOS2 exhibited significantly higher endothelium-adhesion capacity compared to KHOS (Figure 2A).

To determine directly whether VWF expression by cancer cells contributes to enhanced adhesion to an EC monolayer, VWF expression in SAOS2, and in malignant glioma U251, was knocked down by specific siRNA followed by the EC adhesion assay. Similar analysis was also performed in KHOS cells that exogenously expressed VWF. Western blot analyses demonstrated effective silencing of VWF expression with VWF-specific siRNA (VWFsiRNA) but not control siRNA (NSsiRNA); as well as exogenous expression of VWF by a lentivirus vector containing VWF cDNA under the regulation of the CMV promoter (CMV-VWF), but not control lentivirus (CMV-GFP containing GFP under regulation of the CMV promoter) (Figure 2B). EC adhesion assays of these VWF knocked down and exogenously expressing cells demonstrated that there were significant reductions in endothelial monolayer adhesiveness of SAOS2 and U251 cells as a result of VWF knockdown (Figure 2C and 2D). In contrast, exogenous expression of VWF in KHOS lead to its increased adhesiveness to an endothelial monolayer (Figure 2E).

**Figure 3:**
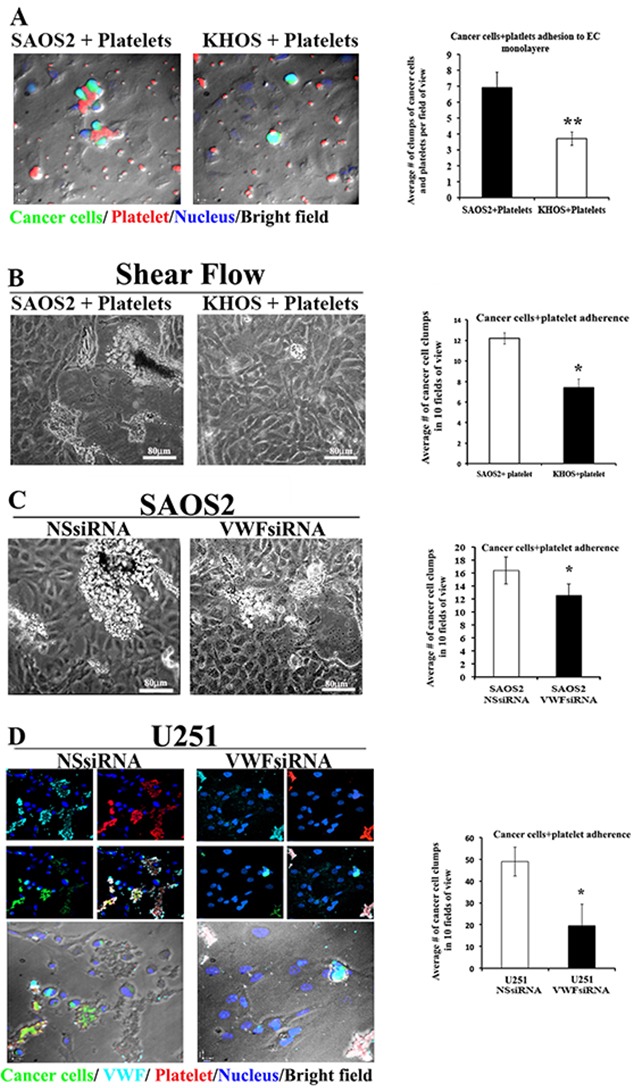
VWF expression increases the adhesion capacity of cancer cells to platelets and endothelial monolayer under static and flow conditions **(A-B)** Fluorescently labelled KHOS and SAOS2 cells (green) were mixed with freshly isolated platelets and maintained for 20 mins. prior to incubation on the monolayer of HUVECs. Adherent cells were fixed and stained using an antibody to CD42b, a marker for activated platelets. Representative photomicrographs of cancer cells (green) -platelets (red) aggregates formation on top of the monolayer of HUVECs are shown. Blue shows nuclei stained with DAPI, which are mostly representative of endothelial cells, since green fluorescence of cancer cells overshadows the blue DAPI staining of their nuclei, and platelets are devoid of nuclei. Clumps of cancer cells and platelets (heteroaggregates) were counted in 5 fields of view and presented as a bar graph. **(B-D)** Using parallel-plate laminar flow adhesion assays, fluorescently labelled cancer cells (KHOS, SAOS2, U251, as well as those treated with NSsiRNA or VWFsiRNA) that were incubated with freshly isolated platelets were perfused over the HUVEC monolayer at shear flows of 1 dyne/cm2 for10 min. **(B and C)** Representative bright field views of cancer cell–platelet heteroaggregate clumps formations for KHOS and SAOS2, untreated and treated with siRNA, are shown. Heteroaggregates clumps were quantified in 10 fields of view for each and presented in bar graphs. **(D)** Immunofluorescent stainings of cancer cell-platelet heteroaggregates on endothelial monolayers marking the presence of cancer cells (green), platelets (red) and VWF (light blue) within heteroaggregates of siRNAs treated U251 cancer cells. Upper panels show individual staining, as well as merged; and lower panels show overlay of merged immunostainings on bright fields. Heteroaggregates clumps were quantified as described for **(B and C)** Results for adhesion assays are representative of minimum of 3 independent experiments (triplicate each) for both static and shear flow adhesion assays.

To test the hypothesis that VWF expression by cancer cells may also contribute to enhanced cancer cell-platelet aggregate formation and increased association of heteroaggregates with an endothelial monolayer, we incubated fluorescently labeled SAOS2 and KHOS cells (using CellTracker™ Green) with freshly isolated platelets for 20 minutes prior to perfusion on a monolayer of HUVECs. After 30 minutes, ECs were washed, fixed and immunofluorescence staining was performed for the activated platelet marker CD42b. Adherent cancer cells were quantified demonstrating that more SAOS2–platelet mixtures adhered to the monolayer of ECs compared to the KHOS-platelet mixtures (Figure 3A).

To explore the adhesion ability of cancer cells under more physiological conditions, we induced shear flow and permitted cancer cell-platelet mixtures to flow over the endothelial monolayer for 10 minutes, using a laminar shear flow chamber. For these analyses we used KHOS, SAOS2 and U251that were either untreated or treated with siRNA. We observed that under shear flow conditions, SAOS2 cells form clumps (cancer cell-platelet heteroaggregates) that are larger in size and number compared to those formed by KHOS (Figure 3B). Similarly, significant numbers of cancer cell-platelet heteroaggregates were observed when U251 cells were used in these analyses. Treatment of SAOS2 and U251 with VWF-specific siRNA (VWFsiRNA) resulted in a significant decrease in numbers as well as the size of the clumps compared to cells that were treated with non-specific siRNA (NSsiRNA) (Figure 3C and 3D). These results support the role of VWF expression by cancer cells to promote their interaction with platelets and adherence to ECs.

### Functional consequences of VWF expression by cancer cells regarding transmigration

**Figure 4:**
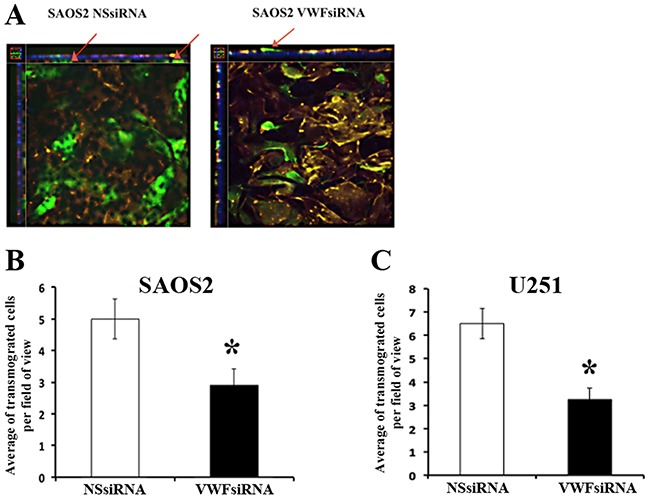
VWF expressing cancer cells demonstrate increased transmigration Transwell assay analyses were performed to determine transmigration capacity of SAOS2 and U251 cells that were treated with NSsiRNA or VWFsiRNA. Cells were incubated on the intact monolayer of HUVECs for two hours and cancer cells (green) that were able to transmigrate through endothelial cell monolayer (red) and move to other side of the membrane (blue) were quantified. **(A)** Representative confocal microscopy images showing cancer cells that either transmigrated (under the blue membrane shown by arrows in NSsiRNA treated SAOS2) or not (above the blue membrane shown by arrow in VWFsiRNA treated SAOS2). **(B and C)** Quantification of transmigrated cancer cells. Results are averages of 3 independent experiments (triplicate each).

To determine whether increased endothelial-adhesiveness of VWF-expressing cancer cells leads to increased transmigration across an endothelial barrier, we performed the transwell migration assay as described in Methods. For these analyses SAOS2 and U251 cells were transduced with a GFP expressing lentivirus and treated with either NSsiRNA or VWFsiRNA, followed by seeding on a monolayer of HUVECs. Following incubation, the transwell membranes containing the endothelial monolayers and cancer cells were subjected to immunofluorescence analyses and confocal microscopy to detect and quantify the number of cancer cells that had transmigrated through the endothelial monolayer. A significantly higher number of VWF expressing cancer cells (NSsiRNA treated cells) transmigrated through the endothelial barrier compared to cells in which VWF was knocked down by VWFsiRNA treatment (Figure 4), demonstrating that VWF expression enhances transmigration of cancer cells.

### VWF expression promotes cancer cell extravasation

**Figure 5:**
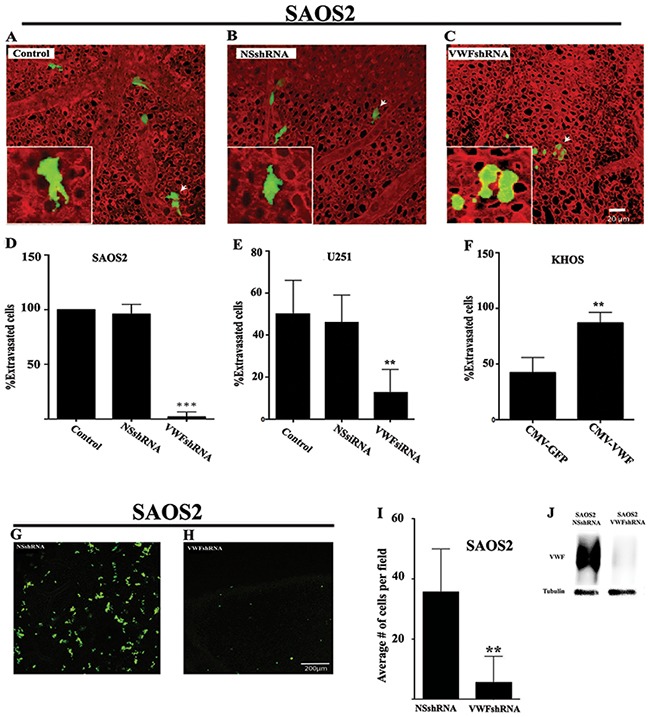
VWF expression by cancer cells results in enhanced extravasation GFP expressing SAOS2 were stably transfected with shRNA targeted specifically against VWF (VWFshRNA) or a non-specific shRNA (NSshRNA). **(J)** Western blot analysis confirmed VWF knockdown in SAOS2 cells expressing VWFshRNA. **(A-D)** Control (NSshRNA) and VWF knockdown (VWFshRNA) SAOS2 cells were injected intravenously into the veins of 12 dpf (12 day post fertilization) chick embryos. Vasculatures were labeled with Rhodamine-Lectin (red) and cancer cells (green) were quantified for their extra- or intravascular localization. Representative images of **(A)** control (untreated GFP expressing SAOS2), **(B)** NSshRNA and **(C)** VWFshRNA transfected SAOS2 are shown. Panels (A-C) show a 3D rendering of the cells and the vasculature. Insets show single optical sections of selected (white arrows) cells. **(D)** Quantification (n=6) of percentage of extravasated control, NSshRNA and VWFshRNA expressing SAOS2. **(E)** Similar CAM assays were performed on glioma cells that were either untreated, treated with NSsiRNA, or VWFsiRNA and the result of quantification (n=6) is shown. **(F)** CAM assays and quantifications (n=6) were performed on KHOS cells that were either transduced with control CMV-GFP or VWF expressing CMV-VWF lentiviruses as described above for SAOS2 and U251 cells. **(G-I)** GFP expressing SAOS2 that were transfected with NSshRNA or VWFshRNA were injected into the tail vein of immunodeficient mice and cancer cell localization in the lung was determined after 24 hours. Representative images of mouse lungs with GFP positive cancer cells (green) are shown in **(G and H)** Quantifications of extravasated cancer cells in the lungs of mice (n=4) are shown in I.

To determine whether the increased endothelial adhesion and transmigration activity of VWF-expressing cancer cells translates into an increased potential for extravasation, we performed the *ex ovo* (chorioallantoic membrane) assay. For these analyses, SAOS2-GFP and U251-GFP cells that were untreated, treated to knock down VWF (VWF siRNA or shRNA), or treated with non-specific interfering RNA (NSshRNA or NSsiRNA) were used. A similar assay was performed on KHOS cells that were transduced with either CMV-GFP or CMV-VWF lentiviral vectors. Cancer cells were injected into the vitelline vein of *ex ovo* chicken embryos. 4-8 hours post injection the embryonic vasculature was labeled with Rhodamine-Lectin and cancer cell extravasation was determined using intravital microscopy analysis as previously reported [23]. SAOS2-GFP cells that were either untreated or treated with NSshRNA migrated intravascularly and passed through the endothelium to embed within the extravascular stroma and proliferate. However, SAOS2-GFP cells treated with VWFshRNA remained trapped inside the vessels with significantly reduced extravasated cells (Figure 5A-5D). Analyses of control and siRNA treated U251-GFP cells demonstrated similar results, with VWFsiRNA treated cells demonstrating significantly fewer extravasated cells compared to control untreated or NSsiRNA treated cells (Figure 5E). Exogenous expression of VWF in KHOS cells after CMV-VWF lentivirus transduction significantly enhanced the extravasation potential of these cells compared to KHOS that were transduced with control CMV-GFP lentivirus (Figure 5F).

Further support for the hypothesis that VWF expression confers a significantly enhanced extravasation potential to cancer cells was provided by our *in vivo* mouse extravasation experiments, in which SAOS2-GFP expressing cells, treated with either VWFshRNA or NSshRNA, were injected into the tail vein of immunodeficient mice, and using an immunofluorescence imaging, the lungs of injected mice were analyzed for the presence of cancer cells 24 hours post injection. The quantity of SAOS2 treated with NSshRNA was significantly higher than those treated with VWFshRNA in the lungs of injected mice (Figure 5G-5I). Taken together, our *ex ovo* and *in vivo* experiments strongly support the hypothesis that acquisition of VWF expression confers an enhanced extravasation capability to cancer cells.

### Mechanism of *de novo* activation of VWF expression in cancer cells

For exploration of the mechanism of VWF transcriptional activation in cancer cells, we chose to focus on the two osteosarcoma cell lines to provide a pair of VWF expressing (SAOS2) and non-expressing (KHOS) cancer cell lines with similar origin (both osteosarcoma) for comparative analyses. Usually VWF expression is exclusively restricted to ECs and megakaryocytes, so we first explored whether expression of VWF in SAOS2 is indicative of the acquisition of an endothelial cell phenotype. Towards this goal we explored the expression pattern of other endothelial-specific genes in the SAOS2 and KHOS. Except for VWF, other endothelial cell-specific genes that were analyzed were either not detected or were similarly expressed in the two cell types (Supplementary Figure 1 Supplementary Data). These results suggest that VWF expression by SAOS2 cells is not a consequence of a general phenotypic shift of these cells towards acquiring an endothelial cell phenotype. Acquiring VWF expression by cancer cells of a non-endothelial/megakaryocyte origin suggests an alteration in the gene regulatory mechanisms that should otherwise inhibit VWF expression in these cells.

**Figure 6:**
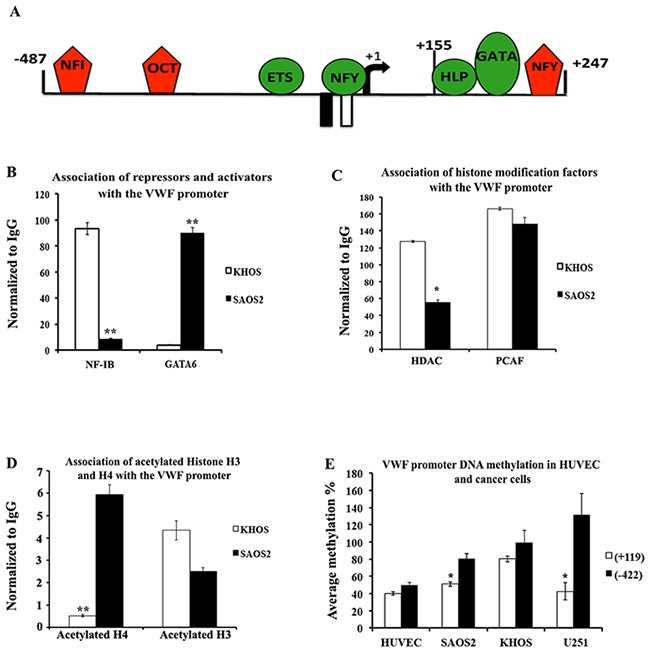
Transcription factor associations and epigenetic modifications of the VWF promoter in VWF-expressing and -non-expressing cancer cells **(A)** Schematic representation of the VWF promoter and transcription factors that positively (green) and negatively (red) regulate the promoter activity. **(B-D)** Chromatin immunoprecipitation (ChIP) analyses of SAOS2 and KHOS were performed (n=3 for each factor) to determine association of **(B)** NF-IB and GATA6, **(C)** histone modifying cofactors PCAF and HDAC1, and **(D)** acetylated histones H3 and H4. **(E)** DNA isolated from cancer cells (SAOS2, KHOS, and malignant glioma U251) and HUVEC (as a positive control for VWF expressing ECS) were subjected to digestion with methylation sensitive restriction enzymes and subjected to quantitative-PCR analyses (using VWF promoter specific primers as described in Methods) to determine relative methylation status of the CG nucleotides at positions -422 and +119. The results are averages of 5 independent experiments.

Previous analyses of the transcriptional regulation of the VWF gene have demonstrated participation of a number of activators (GATA trans-acting factors, Ets, Histone H1-like protein, NFY interacting with CCAAT elements) and repressors (NF-I, Oct1, NFY interacting with a non-consensus sequence) (Figure 6A), as well as chromatin modifications and DNA methylation [24–35]. To explore the mechanism of transcriptional activation of the VWF gene in cancer cells, we explored the presence and VWF-chromatin binding pattern of these regulatory trans-acting factors on VWF-expressing (SAOS2) and non-expressing (KHOS) osteosarcoma cell lines. Comparative RNA analyses demonstrated that the expression level of trans-acting factors, which function as activators of VWF (Ets, GATA2 and GATA6), were not significantly different between the two cell types. The expression levels for two of the subunits that comprise nuclear factor Y (NFY), namely NF-YA and C were not altered, while the levels for the other subunit NF-YB were increased in SAOS2 compared to KHOS (Supplementary Figure 2A Supplementary Data). Since NFY can function either as an activator or repressor of VWF, we could not determine the correlation of increased NF-YB levels to VWF expression; however, similar levels of expression of other factors that were shown to strictly function as activators in the two cell types suggests that acquiring VWF expression does not generally correlate with increased levels of trans-acting factors that function as activators of VWF promoter. Analyses of the expression levels of various isoforms of NF-I transacting factor that strictly function as a repressor of VWF promoter demonstrated that while NF-IA, C and X were expressed at higher levels in SAOS2, NF-IB levels were significantly decreased in SAOS2 compared to KHOS (Supplementary Figure 2B and 2C Supplementary Data). These results suggest that decreased levels of the repressor NF-IB might correlate with increased VWF expression in SAOS2.

To determine whether increased VWF expression correlates with altered association of these transcription factors with the VWF promoter, we performed chromatin immunoprecipitation (ChIP) assays. Consistent with lower levels of NF-IB in SAOS2, ChIP analyses also demonstrated a significant decrease in binding of NF-IB to the VWF regulatory sequences in SAOS2 compared to KHOS (Figure 6B). Using a pan NF-I antibody that does not discriminate among the four isoforms of NF-I (A, B, C and X), ChIP analyses showed that, in general, NF-I interaction with the VWF promoter was significantly reduced in SAOS2 compared to KHOS (data not shown). These results suggest that despite higher levels of NF-IA, -C and -X in SAOS2, NF-I interaction with the VWF promoter in SAOS2 is significantly reduced compared to KHOS. ChIP analyses of GATA6 association with the VWF promoter demonstrated that despite similar levels of this trans-acting factor in the two cell types, significantly higher levels of GATA6 were associated with the VWF promoter in SAOS2 compared to KHOS. The results demonstrated that VWF expression in SAOS2 was associated with decreased expression and interaction of the repressor NF-IB, as well as increased association of GATA6 with the VWF promoter, when compared to KHOS (Figure 6B).

To gain further insight into the mechanism of VWF transcription, we determined the correlation of VWF expression with epigenetic modifications of the VWF promoter in SAOS2 and KHOS. We have previously demonstrated that the VWF promoter in endothelial and non-endothelial cells is differentially associated with histone deacetylase (HDACs 1 and 2) and that the net result is increased acetylation of histone H4 that is associated with the active VWF promoter [28] [36]. Thus, to determine whether VWF expression in SAOS2 is associated with similar VWF chromatin modifications, we performed ChIP analyses to determine the association of HDAC, P300/CBP-Associated Factor (PCAF) and acetylated histones H3 and H4 with the VWF chromatin encompassing the promoter region. While the level of PCAF association with the VWF promoter was similar between the two cell types, HDAC association was significantly decreased in SAOS2 compared to KHOS (Figure 6C). Furthermore, association of acetylated histone H4 (but not H3) was significantly higher in SAOS2 compared to KHOS (Figure 6D). These results are consistent with those previously reported for VWF expression in ECs, and demonstrate that acquiring VWF expression in SAOS2 is accompanied by decreased association of HDACs and subsequently increased acetylation of promoter associated histone H4.

To explore the association of VWF expression with the methylation pattern of the VWF promoter sequences, we determined the methylation status of two specific CG elements located at -422 and +119 that were reported to be non-methylated specifically in ECs. Quantitative PCR were performed on the DNA isolated from SAOS2 and KHOS, as well as malignant glioma U251 (a glioma cell line that also expresses VWF) and HUVECs, subjected to digestion by methylation sensitive restriction enzymes. Comparison of the methylation status of the -422 site demonstrated that all three cancer cell types (SAOS2, KHOS and U251) exhibit increased methylation compared to HUVECs (Figure 6E). However, when comparing KHOS and SAOS2, there were no significant differences in methylation status of the -422 sites between these two cell types. In contrast, site +119 exhibited significantly less methylation in SAOS2 compared to KHOS (Figure 6E). Methylation levels for this site were similar to those observed in HUVECs. VWF expressing U251 malignant glioma cells also exhibited methylation levels at the +119 site that were similar to HUVEC (Figure 6E). These results are consistent with decreased methylation, specifically at site +119, being associated with the VWF promoter activation in cells that express VWF.

### Sub-populations of cancer cells in patients with osteosarcoma and glioma tumors express VWF

**Figure 7:**
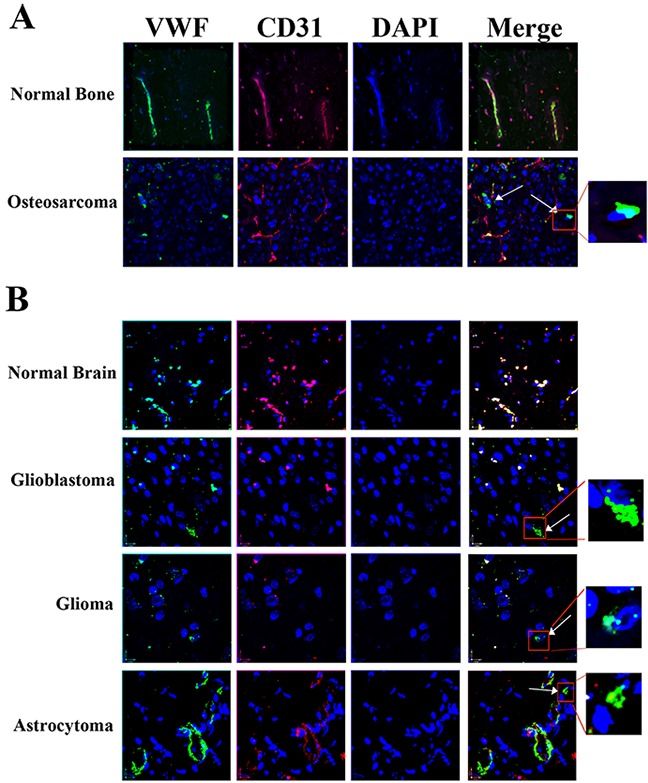
Detection of VWF expressing cancer cells in osteosarcoma and glioma patient tumor biopsies Confocal microscopy and immunofluorescent staining for VWF (green) and endothelial cell specific protein CD31 (red) were performed in **(A)** normal bone, and an osteosarcoma tumor sample; **(B)** Normal brain, a glioblastoma, a glioma and an astrocytoma tumor samples. Colocalization of VWF and CD31 (yellow) is shown in all samples, demonstrating VWF expression by vascular ECs in all samples. However, in tumor samples, but not normal controls, some VWF expressing cells were also detected that did not exhibit co-localization with CD31, supporting their non-endothelial origin (shown by arrows and the enlarged region inside the red square shown). Blue represents DAPI stained nuclei. Results are representative of two independent immunostaining for each sample.

**Figure 8:**
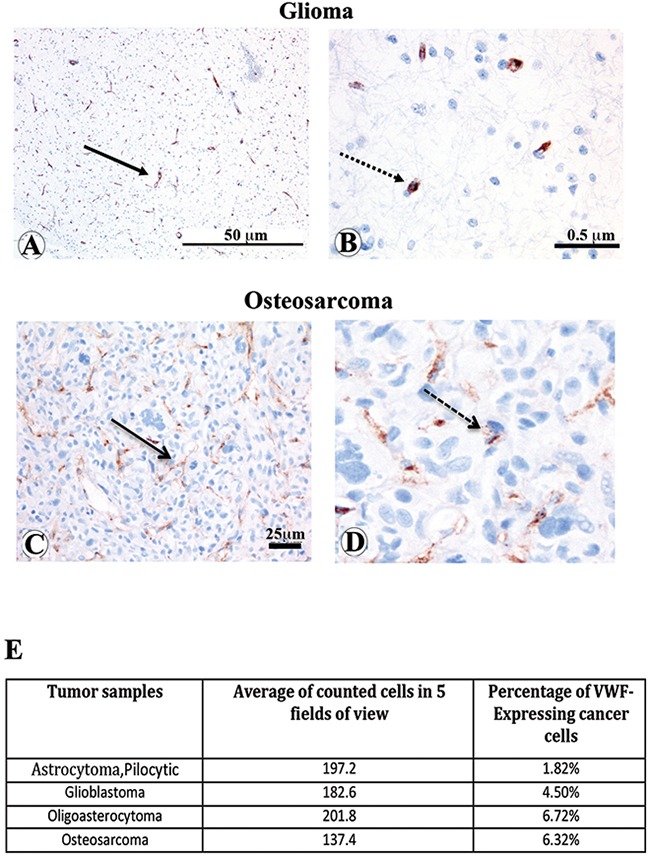
Immunohistochemistry staining and quantification of VWF expressing cancer cells in osteosarcoma and glioma patient tumor biopsies Immunohistochemical (IHC) analyses of tumor samples described in Figure 7 were performed to detect and quantify VWF expressing cancer cells. Representative IHC stainings of **(A** and **B)** malignant glioma and **(C** and **D)** osteosarcoma biopsies with VWF expression localized to vasculature (representative vessels shown by solid arrow) and tumor cells (representative shown by dashed arrow) are shown. The VWF expressing glioma cancer cells in panel **(D)** (5x enlargement of panel **C**) is shown. **(E)** VWF expressing cancer cells in each tumor biopsy was identified and quantified as percentage of VWF expressing cancer cells per total number of cells in a field of view.

To further probe the physiological relevance of our *in vitro* and *in vivo* results regarding the functional consequences of VWF expression by cancer cells, we analyzed one patient-derived osteosarcoma and three separate brain tumors for VWF expression. We performed immunofluorescence staining on tumor samples as well as normal brain and bone control tissues using antibodies against the endothelial marker CD31 and VWF (Figure 7). In the normal brain and bone tissues all cells that express VWF (green), also express CD31 (red), indicating that all the VWF expressing cells detected are of endothelial origin. However, in all tumor samples we observed some cells that only express VWF but not CD31, (shown by white arrows) supporting their non-endothelial origin; this result is consistent with our hypothesis that these tumor cells acquired VWF expression. We also performed immunohistochemistry analyses to detect and quantify VWF expressing cancer cells in these tumor biopsy samples. VWF expressing cancer cells (shown by dashed arrows) were distinguished from VWF expressing vascular endothelial cells (shown by solid arrow) and quantified by an expert pathologist (Figure 8A-8D). The results demonstrated approximately 1.8 to 6.7% VWF expressing cancer cells in these tumor biopsies (Figure 8E).

Although these analyses were performed on only a few tumor samples *in situ*, our results underscore the physiological relevance of endogenous VWF expression by cancer cells since they demonstrate that this phenomenon is not restricted to cancer cell lines grown *in vitro*.

## DISCUSSION

Commonly used expression of VWF as a marker for endothelial cells is reflective of its highly restrictive regulation and exclusive expression in ECs and megakaryocytes. Thus, demonstration of VWF expression in cancer cells, which are neither endothelial nor megakaryocytic in origin, presents a unique opportunity to determine how and why this endothelial specific gene is activated in some cancer cell types. To determine the functional consequences of VWF expression by osteosarcoma and glioma cancer cells, we compared their adhesion ability to EC monolayers under static conditions, shear flow, and in the absence and presence of platelets. Our analyses demonstrated that in all these processes, VWF expression confers an increased ability of cancer cells that express this protein to adhere to endothelial monolayers and platelets, processes that may contribute to an increased metastatic potential. VWF knock down in VWF-expressing cancer cells (osteosarcoma SAOS2 and malignant glioma U251), as well as exogenous VWF expression in non-expressing cancer cells (osteosarcoma KHOS), confirmed that increased adhesive capacity was directly and significantly influenced by VWF expression. Metastatic processes also involve migration and extravasation of cancer cells from the bloodstream and “seeding” of target organs [37, 38]. *In vitro* migration assays of SAOS2 and KHOS demonstrated an increased migration capability for SAOS2 compared to KHOS, which was significantly reduced by VWF knockdown. Furthermore, *in vivo* analyses of the extravasation potential of these cells demonstrated a significantly higher rate of extravasation for SAOS2 compared to KHOS in CAM assays, as well as in the extravasation assay performed in the mouse. Direct involvement of VWF in extravasation was shown by significant reduction as a result of VWF knock down in SAOS2, and significant enhancement as a result of VWF exogenous expression in KHOS. CAM assay of U251 cells demonstrated that in these VWF expressing malignant glioma cells VWF knockdown also significantly reduces extravasation.

Based on these results, we propose that acquiring VWF expression confers a metastatic advantage to subpopulations of cancer cells, through enhancing their platelet interaction, adhesion capacity, migration, and extravasation capability. We propose that this hypothesis may bring together seemingly contradictory evidence regarding the role of VWF (as pro [12, 13] or anti-metastatic [7, 15]) in cancer metastasis. These contradictory observations may be reconciled if we consider a distinct role for VWF when expressed by ECs compared to that acquired by cancer cells. We hypothesize that cancer cells that do not express VWF may be susceptible to the pro-apoptotic effect of exogenously applied VWF. Such cancer cells will have enhanced survival and metastasis if presented to VWF knockout mice. Furthermore, if a population of cancer cells expresses VWF and this contributes to their metastatic role, they will continue to metastasize in the VWF knockout mice. However, in experiments in which anti-VWF antibodies were introduced in mice, these antibodies will exert their effect not only on VWF from endothelial and megakaryocytes, but also on the subpopulation of cancer cells that express VWF. Thus, interfering with VWF function in VWF positive cancer cells may be the mechanism by which the metastatic process was affected in experiments that involved anti-VWF antibody.

We explored the mechanism by which VWF expression is acquired in cancer cells. Previous analyses of VWF transcriptional regulation identified a number of *cis*- and trans-acting factors that regulate VWF transcription. Although an endothelial-specific master regulator has not been identified, several repressors and activators that in combination participate in VWF transcription regulation have been identified [24–30, 33]. In addition, distinct roles for chromatin modifications and DNA methylation were also demonstrated in VWF transcriptional regulation [28, 31, 32]. Based on this information, we explored the role of various components of the VWF transcriptional regulatory system and demonstrated that VWF expression in SAOS2 was associated with significantly decreased binding of the repressor NF-I to the VWF promoter; while binding of the activator GATA6 to the promoter was significantly enhanced. Furthermore, chromatin modifications corresponding to increased levels of acetylated histone H4 associated with the VWF promoter was observed, and DNA methylation associated with a specific region in the promoter was reduced. These results were consistent and similar to what was observed for the active VWF promoter in ECs compared to non-ECs. Analyses of the VWF promoter in a VWF expressing glioma cell line were similar (data not shown). These observations in cell lines with divergent ontologies, i.e. in bone and brain, support distinct upstream and downstream events that converge to regulate VWF gene expression. These mechanisms are similar and target NF-I, GATA6, Histone H4 acetylation and DNA methylation. Our results provide strong evidence for the role of these regulatory components in establishing VWF transcription and present them as potential targets for future development of therapies towards regulation of VWF expression in cancer cells, and interference with the metastatic process.

## MATERIALS AND METHODS

### Cell culture

Human umbilical vein endothelial cells (HUVEC) and HEK293 cells were maintained as previously described [28]. Fibroblast, MDC1 and HeLa cells were grown in DMEM media according to standard protocols [39]. Primary renal Proximal Tubule Epithelial cells (PTEC) were obtained from ATCC and maintained according to the manufacturer's protocol. Osteosarcoma SAOS2 (Lonza), KHOS (Lonza) and malignant glioma [U251and M049 (gift from Dr. R. Godbout)] were cultured using McCoy's 5A (for SAOS2) and DMEM (KHOS and glioma) media according to the Lonza protocol.

### RNA and protein analyses and the chromatin immunoprecipitation (ChIP) assay

Quantitative RT-PCR, Western blot analysis and ChIP assays were performed as previously described [34, 35]. All antibodies were purchased from Abcam Inc. (Abcam, Cambridge, MA, USA), except for VWF (Dako Cat. No. A0082). For Western blot analyses and RT-PCR, lysates from malignant glioma cells (CLA, T98)] and several patient derived glioblastoma cell lines (A4-003 to A4-007) were provided by Dr. R. Godbout.

### Immunohistochemistry and Immunofluorescent staining of cultured cells and tissues

Immunofluorescent staining of cultured cells were performed as previously described [34]. Immunofluorescent staining of slides containing sectioned paraffin imbedded biopsies of normal human brain and glioma (Dr. D. Eisenstat from the Brain Tumor Tissue Bank, London, ON); normal bone (distal humeral resection bone samples, Folio Biosciences, Powell, OH) and osteosarcoma (provided by Dr. C. Sergi) were performed according to standard protocols [40]. Primary antibodies used were CD31 (Dako Omnis, Denmark) and VWF (sheep FTCI pre-conjugated anti-VWF antibody, Abcam-ab8822 and Dako Cat. no: A0082). For Immunohistochemistry analyses tissue sections were incubated with a monoclonal antibody against human VWF (Anti-Von Willebrand Factor antibody [F8/86] (ab778)) at 1:30 dilution at 37 degrees C for 32 min. and automatically stained using a Discovery XT Ventana autostainer following damasking procedures to retrieve antigens. Immunohistochemistry analyses of the above mentioned paraffin embedded tumor biopsy sections followed. The use of patient biopsy samples was approved by the Health Research Ethics Board at the University of Alberta.

### DNA methylation analysis

Two potential methylation sites (cytosines in CpG dinucleotide) located in the VWF promoter (-422 and +119) were tested for methylation using the OneStep qMethyl kit (Zymo Research, Irvine, CA), according to the company's manual.

### Generation of VWF knocked-down and exogenously expressing cancer cells

Transient VWF knockdowns using siRNA were performed as previously described [34]. For these analyses following two distinct sequences of siRNA were used: Hs_VWF_4 [Target Sequence 5′-AACATGGAAGTCAACGTTTAT-3′ (QIAGEN Cat. no.: SI00011830)] and Hs_VWF_2 [Target sequences 5′-ACGGCTTGCACCATTCAGCTA-3′(QIAGEN Cat. no.: SI00011816)]. Stable knock down of VWF were performed using shRNA (sc-36828-v and sc-108080 Santa Cruz Biotechnology, Santa Cruz, CA) in lentiviral vectors according to the manufacturers’ protocol. Cells were also stably transduced with a GFP/luciferase expressing lentivirus (provided by Dr. J. Lewis) and GFP positive cells were selected by fluorescence-activated cell sorting (FACS) analysis. Exogenous expression of VWF and GFP (non-specific control) was achieved by transduction of KHOS cells with -Lentivirus-VWF-GFP and Lentivirus-GFP (Abcam company cat# LVP356569 and cat#LVP690) according to the manufacturer's protocol.

### Cancer cell adhesion to HUVEC monolayer under static and shear flow conditions

Cancer cells were transduced with lentivirus expressing GFP or labelled with cytoplasmic staining reagent (CellTracker™ Green CMFDA Dye- Invitrogen) according to the manufacturer's protocol. In static conditions, 10^5^ cells were incubated on top of a monolayer of HUVEC cells for 30 min, washed with the PBS and trypsinized for FACS analyses based on green fluorescence from CellTracker™ Green or GFP. Similar analyses were performed using cancer cells that were pre-incubated with freshly isolated human platelets for 20 min. Laminar flow adhesion assays were performed using platelet-cancer cell mixtures as previously described [34]. For immunofluorescent analyses non-adherent cells were removed and the slides containing adherent cells and HUVEC monolayers were fixed and subjected to stainings using CD42b specific antibody (BD Pharmingen Cat# 555473) alone (for static condition), or co-staining with VWF- specific antibody (for laminar flow) as previously described [34]. Cancer cells were identified by green fluorescence from GFP expression or cell tracker and DAPI staining identified the cells nuclei.

### The ex ovo chick embryo assay

Cells (10^5^) were injected intravenously into a vein of chick chorioallantoic membrane (CAM). ECs within the CAM were marked by intravenous injection of Lectin-Rhodamine/Fluorescein. Cancer cells were assessed 8 hours (for U251 and SAOS2) and 4 hours (for KHOS) post-injection for their intra- or extravascular localization (extravasated) and quantified as previously described [23].

### Mouse experimental lung extravasation assay

Cancer cells (8.7×10^5^) were injected into the tail vein of immunocompromised mice (NOD *scid* gamma 005557, Jackson Laboratory). Mice were euthanized 24 hours post-injection and lungs were analyzed for extravasated cells by intravital microscopy as previously described [23]. GFP+ cancer cells in the lung were quantified. The Health Sciences Animal Policy and Welfare Committee at the University of Alberta approved all animal housing and experimentation.

### Transwell assay

Membranes in 24 well transwell culture dishes (Corning Transwell, Corning Inc. Corning, NY) were coated with 2% gelatin. HUVECs were grown on the transwell to generate an intact monolayer. Cancer cells (10^4^) were added to the endothelial monolayer and incubated for two hours. Cells were washed and fixed with 4%PFA, then stained for CD31 as an EC marker. Membranes were mounted in mounting media on the slides and ECs were quantified.

### Statistical analyses

Data are given as a mean (SD) and statistical analyses used the paired *t* test. Statistically significant changes (p<0.05) are marked by asterisks (*).

## SUPPLEMENTARY FIGURES


