# Supplementary material for: Functional assessment of von Willebrand factor expression by cancer cells of non-endothelial origin
Source: Oncotarget. 2016 Dec 27;8(8):13015–29. doi: 10.18632/oncotarget.14273 (PMC5355073; doi:10.18632/oncotarget.14273)
Supplement: Supplementary file 1 [file oncotarget-08-13015-s001.pdf]

Functional assessment of von Willebrand factor expression by cancer cells of non-endothelial origin

SUPPLEMENTARY FIGURES

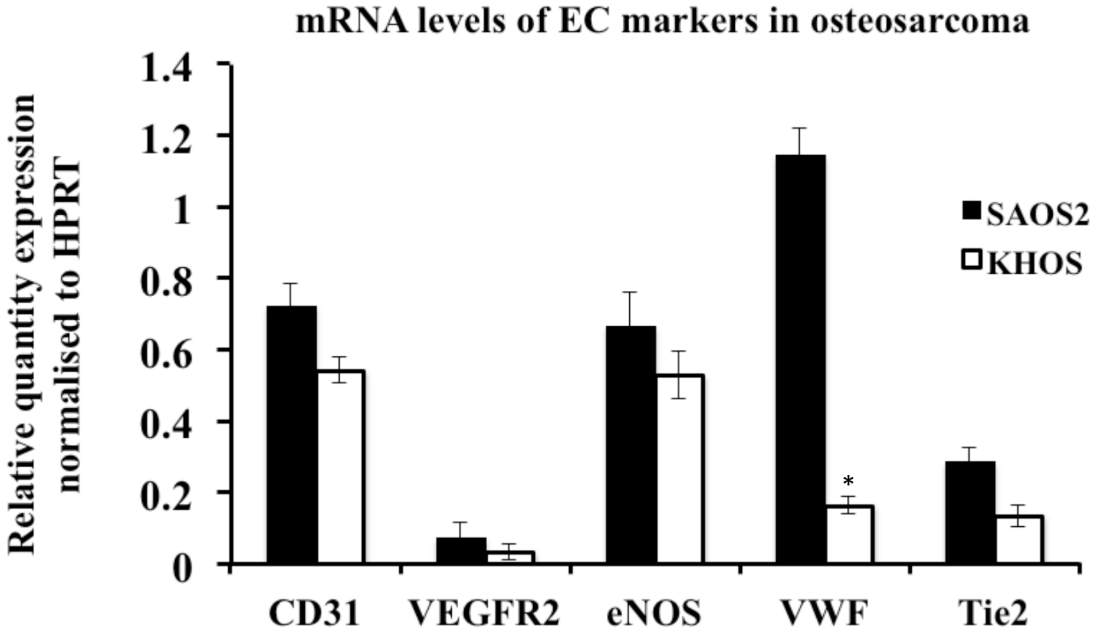

Supplementary Figure 1: The mRNA levels of several endothelial specific genes in osteosarcoma SAOS2 and KHOS cell lines. Quantitative RT-PCR were performed to determine the mRNA levels of CD31, VEGFR2, eNOS, VWF and Tie 2 in the two osteosarcoma cell lines.

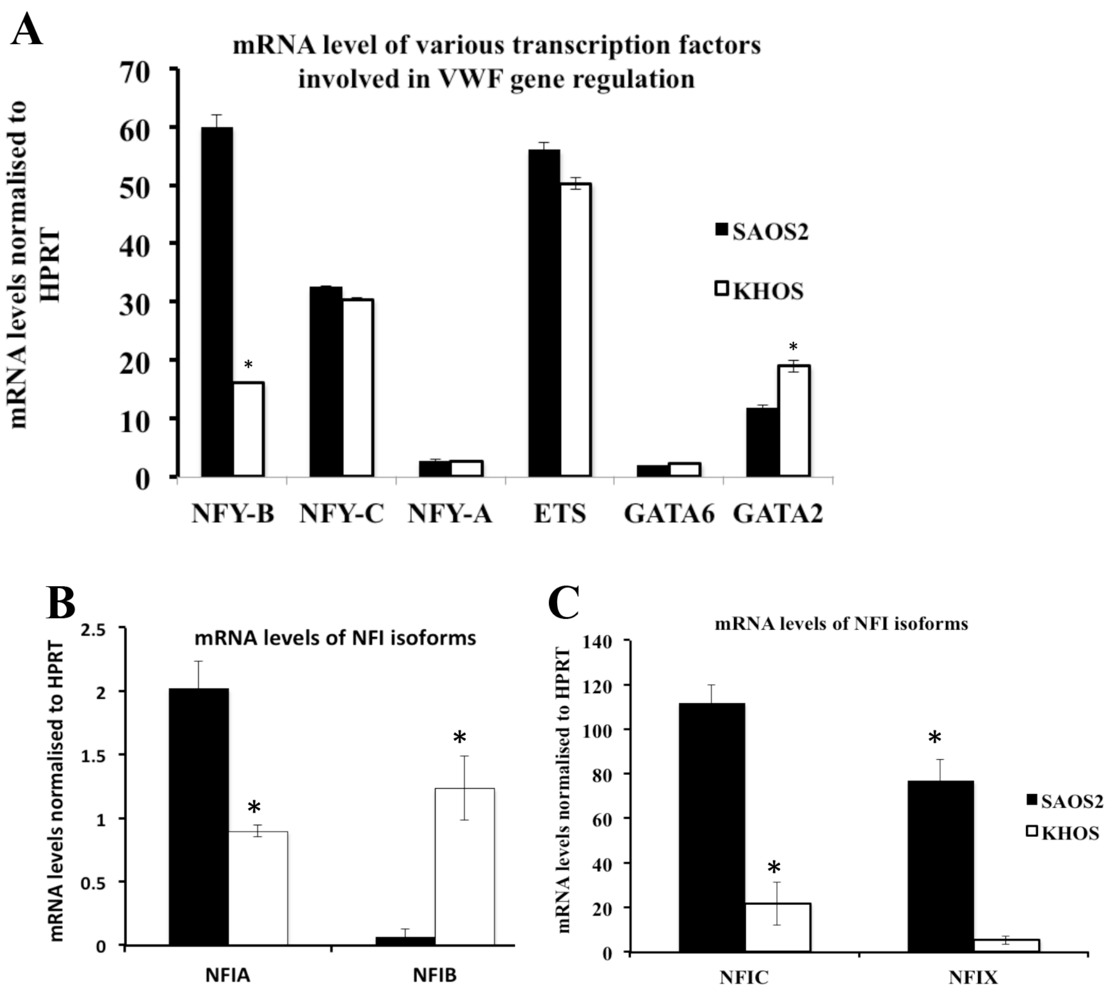

Supplementary Figure 2: The mRNA levels of various transacting factors in osteosarcoma SAOS2 and KHOS cell lines. **(A)** The mRNA levels of NFY subunits (NFY-A, B and C) are shown. NFY functions as both activator and repressor depending on its binding site on the VWF promoter. Also shown are the mRNA levels for ETS, GATA2 and 6 transacting factors which function as activators. **(B** and **C)** The mRNA levels of repressor NFI isoforms A, B, C and X are shown.
